# Investigation and Evaluation of Children’s Blood Lead Levels around a Lead Battery Factory and Influencing Factors

**DOI:** 10.3390/ijerph13060541

**Published:** 2016-05-28

**Authors:** Feng Zhang, Yang Liu, Hengdong Zhang, Yonghong Ban, Jianfeng Wang, Jian Liu, Lixing Zhong, Xianwen Chen, Baoli Zhu

**Affiliations:** 1 Department of Occupational Disease Prevention, Jiangsu Provincial Center for Disease Prevention and Control, Nanjing 210009, China; zhangfeng0401@163.com (F.Z.); zhang-hd@263.net (H.Z.); banyh@jscdc.cn (Y.B.); wjf737@163.com (J.W.); liujiandoc@sina.com (J.L.); zlxzy0666@sina.com (L.Z.); 2 Jiangyin Municipal Center for Disease Prevention and Control, Wuxi 214434, China; liuyangcl@126.com (Y.L.); cxw321523@sina.com (X.C.)

**Keywords:** children, blood lead level, lead battery recycling plan

## Abstract

Lead pollution incidents have occurred frequently in mainland China, which has caused many lead poisoning incidents. This paper took a battery recycling factory as the subject, and focused on measuring the blood lead levels of environmental samples and all the children living around the factory, and analyzed the relationship between them. We collected blood samples from the surrounding residential area, as well as soil, water, vegetables. The atomic absorption method was applied to measure the lead content in these samples. The basic information of the generation procedure, operation type, habit and personal protect equipment was collected by an occupational hygiene investigation. Blood lead levels in 43.12% of the subjects exceeded 100 μg/L. The 50th and the 95th percentiles were 89 μg/L and 232 μg/L for blood lead levels in children, respectively, and the geometric mean was 94 μg/L. Children were stratified into groups by age, gender, parents’ occupation, distance and direction from the recycling plant. The difference of blood lead levels between groups was significant (*p* < 0.05). Four risk factors for elevated blood lead levels were found by logistic regression analysis, including younger age, male, shorter distance from the recycling plant, and parents with at least one working in the recycling plant. The rate of excess lead concentration in water was 6.25%, 6.06% in soil and 44.44% in leaf vegetables, which were all higher than the Chinese environment standards. The shorter the distance to the factory, the higher the value of BLL and lead levels in vegetable and environment samples. The lead level in the environmental samples was higher downwind of the recycling plant.

## 1. Introduction

Lead exposure is linked to cognitive and behavioral deficits, osteoporosis, hypertension and a range of non-specific symptoms [1,2,3]. Previous reports clearly implicated that human red blood cells exposed to lead displayed decreased ATP concentration trends, lower adenylate energy charge value, metabolic and morphological abnormalities [4]. Besides, lead is also a potent neurotoxin, especially detrimental to children’s developing nervous system and this can be modeled in rodents [5,6]. Many children with elevated blood lead levels (BLLs) are asymptomatic [7], but extensive studies have demonstrated that persistent lead exposure results in problems with IQ, cognition and behavior as a result of brain damage in children. Children in low-income communities are more at risk of lead poisoning because of the inadequate control of environment pollution, and iron and calcium deficiencies are the most common phenomena accompanying lead poisoning [8].

In many countries, children are threatened by lead poisoning [9,10], which is widely employed due to industrial and transportation development. In China, there are a total of 330 million children ranging from 0 to 14 years in age, among whom more than 150 million are under threat of lead poisoning [11]. In June 2001, the Chinese government banned the use of lead gasoline across the country. Encouragingly, the proportion of children with high BLL has gradually decreased. The US Centers for Disease Control and Prevention (CDC) had reduced the level of concern for BLL from 100 μg/L to 50 μg/L. Recent studies have shown the possible adverse health effects at BLLs of less than 10 μg/L in children [12]. A recent investigation of 16 cities in China revealed that the average rate of children with BLLs ≥ 100 μg/L was 7.27% (from 2004 to 2008), which was significantly lower than 29.91% in 2001 [13].

Usually, lead enters into children’s blood circulation system via respiratory, gastrointestinal or other routes. According to studies on lead absorption, the predicted bioavailability of ingested soil-borne lead in children ranged from 25% to 41% [13,14], Miranda [2] *et al.* found that once the blood lead concentrations in children were 20–50 μg/L, their abilities to read and calculate—especially the latter—could be affected.

In the present study, we endeavored to elucidate the relationship between BLLs in children and lead levels in polluted water, soil and vegetable samples near a local battery recycling plant. The information on age, sex, parents’ occupation, distance and direction from the recycling plant was used to stratify children’s BLLs. For each stratification variable, we tested whether there was a significant difference between the sub-groups, and a significant correlation between the variable and BLLs.

## 2. Materials and Methods

### 2.1. Sample Collection

**Figure 1:**
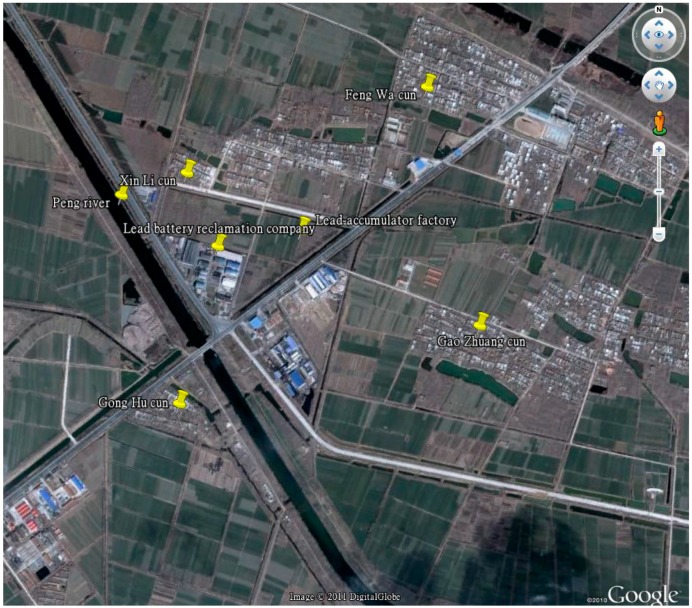
Map of lead battery recycling plant under investigation and sampling sites.

We carried out the project in late 2012 in Yunhe Town (Xuzhou City, Jiangsu Province, China). At the end of 2012, children were reported to suffer from suspected lead poisoning, and we launched an investigation as requested by the local government. During the investigation, a lead battery recycling factory in this town became the focused source of the lead poisoning. We also noted there used to be a lead battery manufacturing factory some 200 m away from a lead battery recycling plant, producing lead-acid batteries. Though the factory had been closed for a long time, its impacts could still be detected in this research, and it was rationally considered a hazard factor. A total of 578 children participated in the recruitment. The principles for subjects’ selection were: (1) children who resided within 1 km of the lead battery recycling factory; (2) the time away from the residence was no more than a month in a year; (3) often have dinner at home; (4) parents did not work in other industries related to lead exposure. Blood samples were selected from 443 children meeting the requirements, after the appropriate consent forms were signed by their guardians. The guardians were interviewed face-to-face by trained personnel, and they also completed a structured questionnaire for information on variables that potentially influence BLL. Accompanied by local officials and under environmentalists’ supervision, we collected water, soil and vegetable samples as initially designed. The locations of the recycling and former manufacturing plant in Yunhe Town are shown in Figure 1 [15].

Thirty-six leafy vegetable samples and forty-nine environmental samples (including 33 soil samples and 16 water samples) were collected from locations within 1 km from the recycling plant. A total of 33 soil samples were collected, including 25 from the top 1–2 cm of the surface and 8 subsurface samples taken at a depth of 3 cm. Four well water samples were collected 2~2.5 m below the surface using a plastic bucket, and 12 canal water samples were obtained from the discharge of the recycling plant and locations upstream and downstream of the recycling plant.

### 2.2. Analysis of Samples

Lead concentrations were determined using an AA Analyst 800 atomic absorption spectrophotometer coupled to a GA-3020 graphite furnace and a programmable sample dispenser (Pekin Elmer Company, Waltham, MA, USA). Linearity was evaluated by calculating the linear correlation coefficient (r), which was >0.9990, and a standard curve was generated for every batch assay. The accuracy was justified using standard addition procedures, and the recovery was between 85% and 115% at different concentrations. Reference material (RM, includes ox blood, laver (seaweed), soil and water) was obtained from the Chinese Reference Material Centre (purchased from the Network of National Information Infrastructure for Reference Materials, Beijing, China) and was measured in the course of every ten-sample analyses. As shown in Table 1, our experimental values are very consistent with the certified recommended values. The between-day precision of lead levels in blood, vegetables, soil and water during the whole course of the study was expressed as relative standard deviation (RSD%). Table 2 shows that both the accuracy and precision of the methods were within acceptable performance criteria. Reagent blanks were applied as blank control, and the lead concentrations in samples were obtained from measurement values minus reagent blanks. Blank levels were all lower than the LOD.

#### 2.2.1. Lead in Blood

Venous blood (5 mL) drawn from each subject were trickled into a heparin anticoagulation tube and stored at 4 °C for lead analysis. Samples were prepared according to the Chinese government standard (WS/T174-1999). Briefly, blood (100 μL) was mixed with 1% Triton X-100 (200 mL, J.T. Baker, Mallinckrodt Baker Inc., Phillipsburg, NJ, USA) and 1% HNO_3_ (200 μL). After vortexing for 30 min, the samples were centrifuged for 4 min at 12,000 rpm. The total sampling volume was 6 μL, which included 3 μL of centrifugate and 3 μL was 1% (*w*/*v*) ammonium dihydrogen phosphate (NH_4_H_2_PO_4_) added as a modifier.

#### 2.2.2. Lead in Vegetables

The vegetable samples were dried at 105 °C to achieve constant weight and further pulverized for a uniform powder with a clean and dried laboratory mill. Sub-samples of 2 g were placed in a crucible, pre-ashed in a hot plate at about 450–500 °C in a muffle furnace. The ashed samples were placed in a 100 mL volumetric flask with 10% of HNO_3_ solution (about 10 mL) and diluted to 100 mL with distilled water.

#### 2.2.3. Lead in Soil

The soil samples were dried at 105 °C until they were of constant weight, crushed and sieved through a 2-mm sieve to remove gravel. Sub-samples (1 g) were placed in a crucible and few drops of distilled water were added to prevent sputtering. The ethylenediamine tetraacetic acid extraction method was used for extracting lead from the soil samples. The extract was then diluted to 100 mL with distilled water and the concentration of lead in it was determined by atomic absorption spectrophotometry.

#### 2.2.4. Lead in Water

Water from two different sources, well and canal, was collected or sampled. The canal water samples were filtered through a 0.8-μm microporous membrane, while well water samples needed no pre-treatment. Each 10 mL water sample was placed in a 20-mL tube, and mixed thoroughly with 0.1 mL of Mg(NO_3_)_2_ (50 μg/L) and 1.0 mL of NH_4_H_2_PO_3_ (120 g/L).

### 2.3. Statistical Analysis

We categorized subjects into different groups according to age, gender, distance and direction from the recycling plant, and parents’ occupation of children. A total of 443 children (260 males and 183 females) aged from 0 to 15 years old (mean = 6.28 ± 4.20 years) were divided into five groups (0–1, 1–4, 4–7, 7–10, and 10–15), respectively, with 37, 119, 107, 91, and 89 subjects in each group. On the basis of distance from the recycling plant, subjects were categorized into four groups (0–250 m, 250–500 m, 500–800 m and 800–1000 m), and each group included 60, 165, 48 and 170 subjects respectively. From the recycling plant, there were 92 children in the northeast zone, 89 in the southeast, 86 in the southwest and 176 in the northwest. Of these children, 140 came from families with their fathers (127) or/and mothers (29) working in a recycling plant. Among 85 vegetable and environmental samples, 22 were collected in the northeast zone, 20 in the southeast, 21 in the southwest, and 22 in the northwest.

The lead content in each group was described in terms of percentiles (P5, P25, P50, P75 and P95), and median. The comparison of over standard rate was conducted with chi-square test. Kruskal-Wallis Test (K-W Test) was employed to evaluate BLLs and the lead levels in vegetable and environmental samples between different groups. The correlation analyses were performed by applying Spearman’s test to discover the correlation of distance between the recycling plant and the blood lead. The multiple regression (MLR) was used to evaluate the relative weight of different independent variables, using the SPSS 15.0 for Windows program (SPSS Inc. Chicago, IL, USA).

## 3. Results

### 3.1. Blood Lead

**Figure 2:**
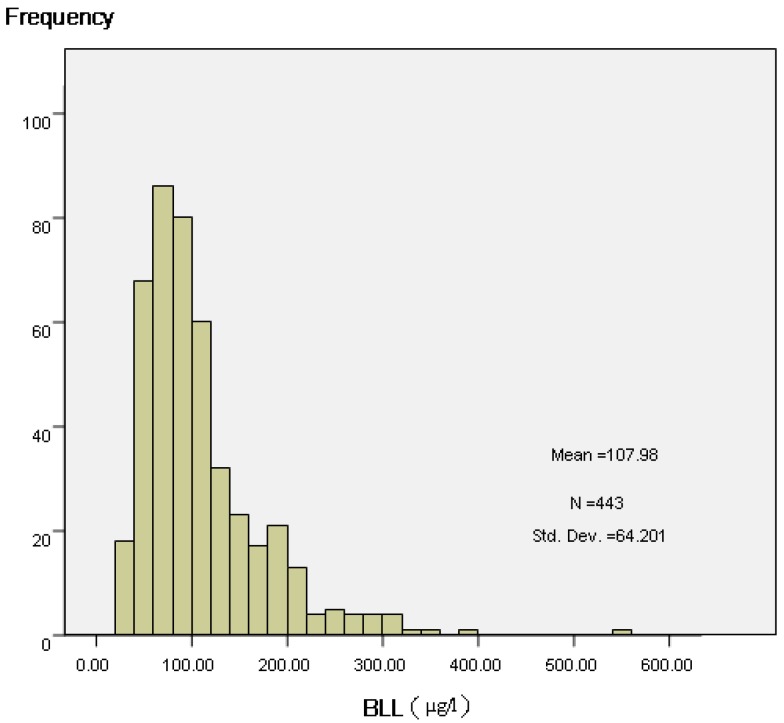
Frequency distribution of children’s BLL (a: measurement data).

Different median and percentiles were observed in different groups. For the total 443 blood samples, the geometric mean (GM) was 94 μg/L, and the median was 89 μg/L. BLLs in children expressed as P5-P95 were 42–232 μg/L. 43.12% of the blood lead samples exceeded the Chinese Ministry of Health diagnosis standard for children lead poisoning at levels greater than 100 μg/L. In addition, 8% of them exceeded 200 μg/L. Information on the distribution of blood lead samples was shown in Table 3. The distribution of BLLs is shown in Figure 2.

Significant differences existed in the percent of children exceeding 100 μg/L among different age groups (χ^2^ = 19.945, *p* = 0.001), and 1–4 year-old children taking the highest percent (57.98%). Females had a significantly lower GM of BLLs compared with males (85 ± 1.7 μg/L *vs.* 100 ± 1.7 μg/L, K-W Test, χ^2^ = 11.974, *p* = 0.001). Moreover, children with at least one parent working in a lead recycling plant had GM of BLLs at levels greater than those who neither of their parents working in that factory (106 ± 1.8 μg/L *vs.* 89 ± 1.8 μg/L, K-W Test, χ^2^ = 28.168, *p* = 0.000).

Distance and direction from the investigated recycling plant were also investigated as influence factors. It was clear that blood levels increased as distance from the recycling plant decreased, and the Spearman’s test illustrated that BLL was negatively correlated with distance from recycling plants (Spearman correlation coefficient = −0.426, *p* = 0.000). Children were stratified into four zones according to distance from the recycling plant. The results indicated a statistically significant consequence among these groups the higher BLL in zones the closer to the plant (K-W Test, χ^2^ = 36.780, *p* = 0.000). We also examined the potential differences among four geographic zones delineated by the direction from the plant. These results showed statistically significant differences (K-W Test, χ^2^ = 29.251, *p* = 0.000) in GM of BLLs between the northwest zone and the other zones, which may due to the distribution of lead emissions from the prevailing winds.

The highest blood lead (556 μg/L) was demonstrated in a 1 year-old female group with father worked in the lead battery reclamation factory and lived 140 m NW of the factory. But which factor played a more important role in the blood lead concentration? The MLR model accounted for up to 50% of the total variance (Table 4), four variables appeared to be significant predictors of the level of blood lead. It was obviously revealed that the most important contributors to the statistical model were the jobs of the parents and, at a lower level, the distance from the factory.

### 3.2. Lead in the Environment and Vegetable

Results of lead from the water, soil and vegetable samples were displayed in Table 5, Table 6 and Table 7. One water sample collected from the canal 200 m, the northeast (upstream) of the plant, exceeded Chinese national standard of 50 μg/L for drink water. Lead levels of both canal and well water samples taken within 250 m of the recycling plant were higher than those samples collected out of 250 m (K-W test, χ^2^ = 4.216, *p* = 0.007).

Following the report of Wierzbicka *et al.* [16], almost all the lead (97.6%) were accumulated in an insoluble form and that thiols were the metal complexing compounds of lead. The percent excess lead of 36 vegetable samples was 44.44% (the standard is <0.3 mg/kg). Lead levels in all eight vegetable samples exceeded the standard within 100 m from the recycling plant. However, no sample showed lead content exceeded the standard when the distance was more than 800 m. Vegetable lead content was linearly correlated with the distance from the recycling plant, the correlation coefficient = −0.799, *n* = 36, *p* = 0.000. The lead content in vegetable samples from the NW was higher than those from other area, however, the difference was not significant (K-W test, χ^2^ = 1.669, *p* = 0.082).

The percentage of lead content in surface soil with levels above 300 mg/kg was 6.06%, but lead levels in all the subsurface soil samples were lower than 300 mg/kg. The lead concentration increased as the distance from the recycling plant decreased, and a correlation coefficient (*R*^2^ = −0.772, *n* = 33, *p* = 0.000) was calculated. Kruskal-Wallis Test results suggested that lead content in soil samples from the NW group was higher than those from other directions (K-W test, χ^2^ = 5.723, *p* = 0.018).

## 4. Discussion

Although some new studies have re-evaluated the effects of lead, Wilson [17] thought previous studies overestimated the risk of low concentration of lead exposure because of some cofounding in the epidemiology of lead and the authors stated that policy-makers may need to review recent changes to regulations. However harmful of lead exposure has become a global risk for children, especially in developing countries and regions. Recent reports of BLLs in children living in non-polluted and polluted places are shown in Table 8. Commonly, the mean values or GM values of BLLs in children located in polluted places were higher than that of children living in normal environments. Gottesfeld and Pokhrel [18] reviewed studies from 37 developing countries published from 1993 to 2010, and the average BLL of children who lived nearby battery manufacturing and recycling plants was 290 μg/L. However, the mean BLLs of 108 μg/L in our study approximates the BLL (129, 86, and 120 μg/L) reported in the studies published after 2003 [18]. In Jamaica, BLL mean in children 2–12 year-old, living around a lead smelting facility was 251 μg/L. Therefore, lead pollution and elevated BLL in children require increased governmental attention, especially in developing and low-income countries.

There are other reports of blood lead pollution in China [19,20]. Du and Cao [19] investigated BLLs in children living near a lead recycling plant. They found that the mean BLL from exposed and non-exposed groups were 258 ± 98 μg/L and 115 ± 27 μg/L, both of which exceeded the government standard of 100 μg/L. However, in the control area where were no impacts from a lead-related factory, BLLs of ≥100 μg/L were found to be 10.46% in Beijing, China [21].

In our study, the lead battery recycling plant was located in the middle of a residential area. This may be one of the major reasons for the observed excessive BLLs. The map showed a closed lead battery manufacturing factory located 200 m northeast of the lead battery recycling plant. Equipment and lead battery materials were still in on the closed lead battery manufacturing factory site and lead could be discharged by the rain, which might account for the higher lead content in water at this site. By multiple regression, four risks of elevated BLL were found, including younger age, male, shorter distance from the recycling plant, and parents working at the recycling plant. The group of children residing ≤250 m from the recycling plant had the highest rate of excess lead (71.67%) and GM (140 ± 1.8 μg/L).

BLLs of children were associated with their parents’ job [22,23]. The mean BLLs for the 10 control children was significantly different from the mean BLLs for the children of miners (*p* = 0.02). Chiaradia [22] indicated that lead was probably transported into worker’s houses on the clothes, shoes, hair, skin, and so on. Lead dust could be shaken from the clothes, and children of mine employees may be exposed to lead though touching the clothes, skin, hair, or maybe breathing the air contaminated with lead shaken from the clothes. The result was similar to Chiaradia’ report, where parental occupation was identified as a significant factor which influenced children’s BLLs (OR = 0.597, *p* = 0.024). The investigated recycling plant adopted a reduction method for lead recycling. About 30,000 tons of recycled lead could be processed per year, while at the same time 300 tons of waste residues containing lead could be produced. Workers in the recycling plant exposed to lead dust and fume might carry lead on their work clothes. Following the protocol, workers should have separate clothing after work, but generally they didn’t actually change from their working clothes into their own. After contact with their parents or lead brought home from the workshop, the children would be passively exposed to an environment with high lead pollution. All these inappropriate procedures might contribute to the observed elevated BLLs.

Children are at greater risk for elevated BLLs than adults. However, there is conflicting evidence concerning whether risk for elevated BLLs increases or decreases with increasing age in children. Due to hand-to-mouth activity and a higher gastrointestinal absorption rates, the BLLs of young children are expected to be higher than that of older children. In addition, the gastrointestinal absorption for lead decreases with increasing age. However, not all studies have shown younger children at a greater risk for the development of elevated BLLs. The BLLs in 4- to 12-year-old children in Dhaka (Bangladesh) had a positive linear relation with age, showing an increase with aging in these children [24]. In this study, children above 7 years-old have the lowest GM BLLs (GM = 80 μg/L). Beside the above reason, this may be due to the decreased exposure from the polluted environment including soil and air, because if the children were above 7 years-old, most of them went to a school located about 5 km NE from the lead recycling plant.

Like age, the reports of effect of sex on blood lead concentration were also different. The proportion of boys’ BLLs more than 100 μg/L was significantly higher than that of girls’. Compared with female children, male children tend to spend more time outdoors, likely ingesting soil dust contaminated with lead through hands and mouth. Gender was associated with BLLs in our investigation (*p* = 0.024), which was compatible with the studies conducted in Bangladesh [25]. But Nichania [26] reported that gender was not associated with BLLs (*p* = 0.276), and there was no report on the effect of gender on lead absorption. However, because boys usually played outdoors for longer time and are more active, male children would face more risk of lead exposure; this might be the reason for the higher BLLs of boys in our investigation.

Lead in air can diffuse with wind. Therefore, the lead sedimentation in soil and vegetables is possibly much higher in a downwind direction since the probability of lead concentration in the air is higher in such an area [27]. As reported by the weather department of the meteorological administration of Xuzhou City, the local prevailing wind direction is south-east (SE), and lead in the air may flow with the wind and accumulate in the downwind direction. Therefore the lead levels in vegetable and environment samples were higher in a downwind direction, the NW. Lead levels in environmental and vegetable samples might affect the BLL of children. The important principle behind the collection of vegetable, soil and water samples was the direction from the recycling plant in which the children lived. Those children who lived in area with the highest GM, <250 m from the recycling plant, were also found to be exposed to an environment with the highest percent of excess lead.

Vegetables, soil and water could have been contaminated by lead discharged from the recycling plant, causing elevated BLLs when children are exposed or consumed contaminated samples. Distance can contribute to a high BLL and high lead levels in vegetable and environment samples. The nearer the distance to the factory, the higher the BLL values and lead levels in vegetable and environment samples were. Besides, the lead level in the environmental samples is higher downwind of the recycling plant, to the NW.

Lead levels in environmental samples may have affected the BLLs of children [28,29]. Cao [29] analyzed health risks from the exposure of children to lead near a lager coking plant in China. They indicated that vegetables, drinking water and wheat also can be polluted by coking plants, which affects the blood lead levels of children, and furthermore research showed that the coal used in the coking plant is the dominant source of lead pollution in children’s blood.

The important principle behind the collection of vegetable, soil and water samples was the direction from the factory in which the children were living. It was observed that the higher lead content in the environment samples was in the residences of children with higher BLLs. In the investigation, it was found that the majority of local residents eat home-grown vegetables and eggs. Therefore, the lead can enter into children blood through the consumption of high concentrations of lead vegetables, which leads to the increased BLLs in children.

## 5. Conclusions

Different GM and percentiles were observed in different groups. For the total of 443 blood samples, the GM was 94 ± 1.7 μg/L. The BLLs in children expressed as P_5_–P_95_ were 42–232 μg/L. Children grouped by age, sex, distance and direction from the investigated recycling plant, as well as by parents’ occupation, showed differences within each group. Potential influencing variables were age and parents’ jobs, and the latter had the largest contribution to the high BLL. Vegetables, soil and water could have been contaminated by lead discharged from the recycling plant, causing elevated BLL when children were exposed to or consume contaminated samples.

Distance and direction can make a contribution to the high BLL, especially distance. The nearer the children’s houses are to the factory, the higher the values of their blood lead concentrations are. Children living downwind of the recycling plant had higher BLLs than that of who lived upwind like the environmental and vegetable samples. Age plays an important role in the lead exposure and this is reflected in the higher BLLs observed among younger children (1–4 years old) in our study.

In conclusion, our study revealed that age, gender, distance and direction from the investigated recycling plant, as well as by parents’ occupation could be identified as important influencing factors in children BLLs. Further studies with larger sample sizes and diverse geographic regions are expected to confirm our findings.

## Figures and Tables

**Table 1:** Quality Assurance for Lead in Samples. The Reference Material (RM) results.

Name of RM	Certified	Measured
Lead in ox blood		
Level I (μg/L)	96 ± 8	94.52 ± 3.57 μg/L (*n* = 82)
Level II (μg/L)	189 ± 10	185 ± 4.79 μg/L (*n* = 82)
Lead in vegetable (laver) ug/g	0.81 ± 0.03	0.79 ± 0.04 ug/g (*n* = 16)
Lead in soil (mg/kg)	47.4 ± 3.5	46.92 ± 2.18 mg/kg (*n* = 16)
Lead in water (ug/mL)	2.0 ± 0.12	1.98 ± 0.09 ug/ML (*n* = 8)

**Table 2:** Methodology parameters.

Matrix	LOD	Level	Measured	Recovery (%)	RSD%
Blood (μg/L)	0.45	5	4.87 ± 0.42 (*n* = 54)	97.4	4.5
		50	49.17 ± 1.58 (*n* = 54)	98.3	3.2
		150	151.09 ± 10.21 (*n* = 54)	100.7	6.8
Vegetable (laver) (μg/kg)	5.0	50	48.26 ± 2.13 (*n* = 12)	96.5	4.4
		200	197.84 ± 10.94 (*n* = 12)	98.9	5.5
		500	503.21 ± 19.01 (*n* = 12)	100.6	3.8
Soil (μg/kg)	5	50	49.05 ± 2.97 (*n* = 12)	98.1	6.1
		200	198.28 ± 8.75 (*n* = 12)	99.1	4.4
		500	495.87 ± 23.22 (*n* = 12)	99.2	4.7
Water (μg/L)	2.5	20	19.41 ± 1.06 (*n* = 12)	97.0	5.4
		100	98.76 ± 3.05 (*n* = 12)	98.8	3.1
		500	496.28 ± 14.79 (*n* = 12)	99.3	3.0

LOD means limit of determination, RSD means relative standard deviation.

**Table 3:** Concentrations and descriptive statistics for lead in children’s blood.

	No.	Range of Lead Concentration (μg/L)	Median (μg/L)	Percentile (μg/L)					BLL > 100 μg/L Number and Percent (%)
				5th	25th	50th	75th	95th	
Age of subjects									
0–1	37	34–556	92	36	50	92	128	334	18 (48.65)
1–4	119	33–394	111 *	49	78	111	165	292	69 (57.98)
4–7	107	38–315	88	47	65	88	134	252	42 (39.25)
7–10	91	36–221	80	38	59	78	106	155	26 (28.57)
10–15	89	26–224	82	36	56	84	112	205	36 (40.45)
Gender									
male	260	33–394	100	43	68	100	145	234	132 (50.77)
female	183	26–556	81 **	38	59	81	110	238	59 (32.24)
Distance (m)									
0–250	60	36–556	151	53	89	151	209	325	43 (71.67)
250–500	165	34–394	106 **	44	80	106	144	223	89 (53.94)
500–800	48	37–284	81 **	40	54	81	102	194	14 (29.17)
800–1000	170	26–297	73 **	39	56	73	98	148	45 (26.47)
Direction ^†^									
Northwest (NW)	176	36–556	116	53	57	121	182	298	110 (62.5)
Southwest (SW)	86	34–202	82 **	38	63	79	106	180	25 (29.07)
Northeast (NE)	92	26–279	72 **	36	55	71	96	127	20 (21.74)
Southeast (SE)	89	37–246	82 **	43	83	88	129	173	36 (40.45)
Job of parents									
In the recycling plant	140	34–556	101	43	77	101	148	251	72 (51.43)
None in the recycling plant	303	26–294	84 **	42	62	84	118	224	119 (39.27)
Total	443	24–556	89	42	64	89	133	232	191 (43.12)

^†^ Northeast is Fengwa village, Southeast is Gaozhuang village, Southwest is Gonghu village and Northwest is Xinli village. * *p* < 0.05 (K-W Test); ** *p* < 0.01 (K-W Test).

**Table 4:** Determinant of blood lead as assessed by multiple regression analysis.

Variable	Parameter Normal Model (r^2^ = 0.50)		
	β	*t*-Value	*p*-Value
Age	−0.036	−5.0427	0.000
Distance	−0.052	−5.2072	0.000
Direction	0.010	0.7078	0.479
Parents’ job	0.060	2.9332	0.004
Sex	−0.043	−2.2574	0.024

**Table 5:** The result of water samples (μg/L).

Water Samples	Number	Lead Range	Median	Number and Excess Ratio (%) ^†^
Kind				
well	4	<1–5	45	0(0.00)
canal	12	<1–77	7	1(8.33)
Direction				
Northeast	4	17–77	23	1(25.00)
Southeast	3	1–5	2	0(0.00)
Southwest	5	<1–5	1	0.00
Northwest	4	4–12	15	0.00
Distance (m)				
≤250	4	12–77	23	1(25.00)
~500	4	4–17	11	0(0.00)
~800	4	1–5	4	0(0.00)
~1000	4	<1–1	1	0(0.00)
Total	16	<1–77	7	1(6.25)

^†^ Excess is a lead concentration ratio over 50 μg/L for water.

**Table 6:** The results of vegetable samples (mg/kg).

Vegetable Samples	Number	Lead Range	Median	Number and Excess Ratio (%) ^†^
Direction				
Northeast	9	0.006–1.075	0.051	3(33.33)
Southeast	10	0.049–3.079	0.285	4(40.00)
Southwest	8	0.007–1.475	0.172	3(37.5)
Northwest	9	0.071–4.676	1.049	6(66.67)
Distance (m)				
≤250	12	0.045–4.676	1.352	10(83.33)
~500	8	0.009–1.056	0.451	5(62.50)
~800	8	0.008–0.425	0.119	1(12.50)
~1000	8	0.006–0.147	0.052	0(0.00)
Total	36	0.006–4.676	0.265	16(44.44)

^†^ Excess is a lead concentration ratio over 0.3 mg/kg for vegetables.

**Table 7:** The results of soil samples (mg/kg).

Soil Samples	Number	Lead Range	Median	Exceedance and Ratio ^†^
Kind				
Surface	25	10.07–424.73	101.69	2(8.00)
Subsurface	8	13.60–113.64	52.92	0(0.00)
Direction				
Northeast	9	14.38–121.60	57.85	0(0.00)
Southeast	7	13.60–121.45	46.17	0(0.00)
Southwest	8	29.07–182.81	79.52	0(0.00)
Northwest	9	59.67–424.73	120.08	2(22.22)
Distance (m)				
≤250	11	113.64–424.73	121.60	2(18.18)
~500	8	13.61–282.81	74.33	0(0.00)
~800	8	25.60–101.69	63.16	0(0.00)
~1000	6	10.07–41.06	27.47	0(0.00)
Total	33	10.07–424.73	87.96	2(6.06)

^†^ Excess is a lead concentration ratio over 300 mg/kg for soil.

**Table 8:** The BLLs in children in some recent research studies.

Reference	Country	Age of Subject	Dwell Environment	Study Year	No.	Mean (μg/L)
[9]	Jamaica	2−12years	Lead Smelting	-	107	251
[16]	America	<12 years	smelter communities	2002	169	16
				1980	196	236
[26]	India	0−23months	Low socioeconomic	2003–2004	178	72 (GM)
		24−47 months			191	81
		48−71 months			167	83
		72−95 months			158	87
		96−119 months			104	99
		120−143 months			6	83
[30]	Colombia	5−9 years	Lead fishing net sinker, metal melting	2004	189	55
[31]	Brazil	0−12 years	Battery recycling	2002	624	93
[32]	Chile	5−12 years	Lead storage facilities	1998	93	164
[33]	Uruguay	0−14 years	Urban area	2003	107	94
[34]	Romania	8−12 years	Near to battery factory	2006	37 46	32 51
[35]	German	Child	Normal	2003–2006		35
[36]	Australian	0.5~2 years	Normal	2001~2002	156	27 (GM)
		2~3 years		2002~2003	169	24 (GM)
		3~4 years		2003~2004	133	20 (GM)
		4~5 years		2004~2005	117	19 (GM)
		5~7 years		2005~2006	134	17 (GM)
